# A Newly Modified Salter Osteotomy Technique for Treatment of Developmental Dysplasia of Hip That Is Associated with Decrease in Pressure on Femoral Head and Triradiate Cartilage

**DOI:** 10.1155/2019/6021271

**Published:** 2019-02-06

**Authors:** Seyed Mokhtar Esmaeilnejad-Ganji, Seyed Mohammad Reza Esmaeilnejad-Ganji, Mohammad Zamani, Hesam Alitaleshi

**Affiliations:** ^1^Clinical Research Development Center, Shahid Beheshti Hospital, Babol University of Medical Sciences, Babol, Iran; ^2^Department of Orthopedics, Babol University of Medical Sciences, Babol, Iran; ^3^Boston University School of Medicine, Boston, USA; ^4^Student Research Committee, School of Medicine, Babol University of Medical Sciences, Babol, Iran

## Abstract

**Background and Purpose:**

The Salter innominate osteotomy has been an effective method to treat the developmental dysplasia of hip (DDH) over the past decades; however, several postoperative complications and deficiencies were reported. In this study, we evaluated outcome of a newly modified Salter osteotomy in patients presenting with DDH.

**Methods:**

We reviewed retrospectively 76 patients (90 hips) with DDH aged ≥ 18 months, who underwent open reduction and a modified osteotomy by a single surgeon. The distal osteotomy segment of pelvis was shifted anterolaterally in the amount of osteotomy cross-section, but not downwards. The mean age at surgery was 2 years and 11 months (1.5 to 16 years). Femoral shortening was conducted when necessary. The duration of operation varied between 60 and 90 minutes. The mean follow-up was 4 years and one month (range 15 months to 7 years and 9 months). All patients were followed up both clinically (based on the modified MacKay criteria) and radiologically (based on the modified Severin criteria).

**Results:**

Clinically, 94.5% of hips had excellent and good results at final follow-up, and only 5.5% had a fair condition. Radiographically, at the final follow-up 77.8% of hips were grade IA (excellent), 12.2% were grade IB, 6.7% were grade II, and 3.3% were grade III (fair). The preoperative mean acetabular index was 47.85° (41° to 59), which decreased to 17.16° (13° to 22°) immediately after the surgery (p<0.0001) and progressed to 11.24° (7° to 19°) at the final follow-up (p<0.0001). The mean initial postoperative center-edge angle was 30.3° (25° to 42°) significantly improved to 39.1 (31° to 56°) at the final follow-up (p<0.0001). Avascular necrosis of femoral head occurred in 4.4% of hips (4 patients).

**Conclusion:**

The results show that our modified Salter osteotomy is safe and associated with significant benefit for the management of patients suffering from DDH.

## 1. Introduction

Developmental dysplasia of the hip (DDH), or congenital hip dislocation, is one of the most common abnormalities in neonates [1, 2]. If diagnosed early and treated by bracing or casting, it can be improved in most patients with no need for surgery but, if not managed properly, it needs surgery for reduction [3–5]. Although there are different methods for the treatment of DDH, if the patient is not be treated, he/she will not only develop complications—such as limping and osteoarthritis—but also later change to near joints, like the knee and vertebrae [6, 7].

One of the usual and effective methods for the treatment is osteotomy of the pelvic bone, namely, Salter osteotomy which was initially described in 1961 [8]. In this technique, the pelvic bone undergoes osteotomy above the acetabulum, and the distal segment is shifted outwards, forwards, and downwards for appropriate construction of the acetabular roof. Shifting the distal segment downwards, which is the most displacement in this surgery, opens the osteotomy site anterolaterally which is filled with the triangular bone graft removed from the pelvic bone [8]. Shifting the distal segment downwards causes decrease in the acetabular volume cavity and increase in the pressure on the femoral head, leading to increase in the risk of chondrolysis and avascular necrosis (AVN) of the femoral head. Furthermore, there is a possible displacement of the inserted graft in the gap between the distal and proximal segments, leading to displacement of the distal segment upwards and inwards and decrease in or destroying the primary coverage of femoral head [9, 10].

In the present study, we introduced a newly modified osteotomy and open reduction, expecting to result in less postoperative complications and greater efficacy compared with the classic Salter's technique.

## 2. Materials and Methods

### 2.1. Study Population and Location

In this retrospective study, all patients presenting with DDH aged ≥18 months underwent the modified Salter osteotomy and open reduction by the senior author (Seyed Mokhtar Esmaeilnejad-Ganji) were included. All surgeries were performed between 1995 and 2017 in Shahid Beheshti Teaching Hospital, affiliated to Babol University of Medical Sciences, Iran. Patients with teratologic, syndromic, or neuromuscular dislocation were excluded from the study.

### 2.2. Data Collection

All necessary information was obtained using a checklist, including age, sex, duration of surgery, follow-up time, radiographic data (center-edge [CE] angle and acetabular index), and clinical data. All patients were examined clinically by the orthopedic resident under direct supervision of the attending orthopedic surgeon. To calculate the angles in the patients' radiographs, the Digimizer Image Analysis Software (MedCalc Software, Belgium) was used under supervision of the attending orthopedic surgeon. The clinical evaluation of the patients was done by the modified McKay criteria (Table 1) [11] and the radiological assessment was conducted based on the modified Severin criteria (Table 2) [12] in the follow-up. To find the evidence of AVN, the radiographs were assessed and the severity of necrosis was graded according to Bucholz and Ogden classification [13].

### 2.3. Acetabular Index

This index is referred to as the angle between the horizontal line of Hilgenreiner and the line connecting the two edges of the acetabulum on anteroposterior radiograph.

### 2.4. Center-Edge Angle

This angle is formed between a line connecting the lateral edge of acetabulum with femoral head center and a vertical line on femoral head on anteroposterior radiograph and shows the amount of lateral shift and tilt of acetabulum.

### 2.5. Preoperative Protocol

Before the surgery, the families were given all appropriate information about the type of surgery and its outcomes and the necessary measures required before and after the surgery. In the patients with bilateral DDH, a delay of 3-6 months was applied between the procedures.

### 2.6. Surgical Procedure

The surgery began under general anesthesia. When lying supine, a sand bag was placed under the thorax and pelvis on the affected side, so the pelvis was kept up. With an iliofemoral approach and through the tensor fascia latae and sartorius muscles, the iliac wing was exposed at 1/4 of posterior and 3/4 of anterior of the iliac crest to lower than anterior-inferior iliac spine. The sartorius muscle was shifted inwards, with the head of rectus femoris and abdominal muscles and medial wall periosteum of ilium. The tensor fascia latae muscle with iliac apophysis and lateral wall periosteum were shifted laterally as a flap. Lateral cutaneous nerve of thigh was protected if possible. Then the reflected head of rectus was removed from the joint capsule, and the joint capsule was exposed superoanteromedially, down to the iliopsoas muscle tendon and to the lowest point of acetabulum (acetabular ligament). The iliopsoas was then released from its tendon site on lesser trochanter and the capsule was opened longitudinally from the acetabular ligament to the highest point of acetabulum. The acetabular fossa was then cleaned of fibrofatty tissue and the teres ligament, and the acetabular ligament was released. Then, the sciatic notch was exposed behind the ilium and the pelvic osteotomy was conducted by the Gigli saw at the level of anterior-inferior iliac spine (above the acetabulum). The distal segment was shifted anterolaterally as much as the bone flexibility (on pubic symphysis hinge) allowed, as the distal segment was shifted laterally in the amount of the cross-section of osteotomy and anteriorly in the same or less amount of the osteotomy cross-section. In other words, in the amount of the pelvic osteotomy cross-section was added to the acetabular roof anterolaterally. In this situation, there was no gap between the two segments in the coronal plane and the anterolateral corner of the proximal segment was connected with the posteromedial corner of the distal segment. The segments were then easily fixed by 2-3 pins with 2-4 mm diameter.

The femoral head was reduced and its stability was examined. In this situation, if the pressure on the femoral head was high (that is, the femoral head was not reduced easily and softly), the procedure of femoral shortening was conducted from proximal femoral shaft. The amount of femoral bone resection in shortening was calculated based on the amount of the overriding of the distal segment of femoral shaft on the proximal segment after shaft osteotomy in form of easily (without tension and pressure) reduced femoral head in the acetabulum. The shortening technique was done without rotation or varisation, meaning that no any changes were done in the anteversion of the femoral head during shortening, except when anteversion was more than 60 degrees which was decreased as much as not passing the 45 degrees.

After reduction, if the capsule was wrinkled, it was resected in a way that the wrinkle was eliminated. The capsule was not reefed, because reduction was sufficiently stable in a wide range of hip motion following the roofing procedure. The new stability was obvious and objectively detectable in all our patients in the operating room. Iliac wing was resected by 1-20 mm height and 3-5 cm length based on conditions by Gigli saw and was laid down on the raw surface of the distal osteotomy segment. After completely irrigation of the wound, it was closed in three layers and a Hemovac drain was inserted. Finally, a one and one-half spica cast (respectively for the affected and normal sides) was applied in the most suitable position of hip which was associated with the most coverage of femoral head by acetabulum and the least pressure on the femoral head and soft tissue tension. This position resulted from about 45-60 degree flexion, 10-20 degree medial rotation, and 20-30 degree abduction of the hip.

**Figure 1:**
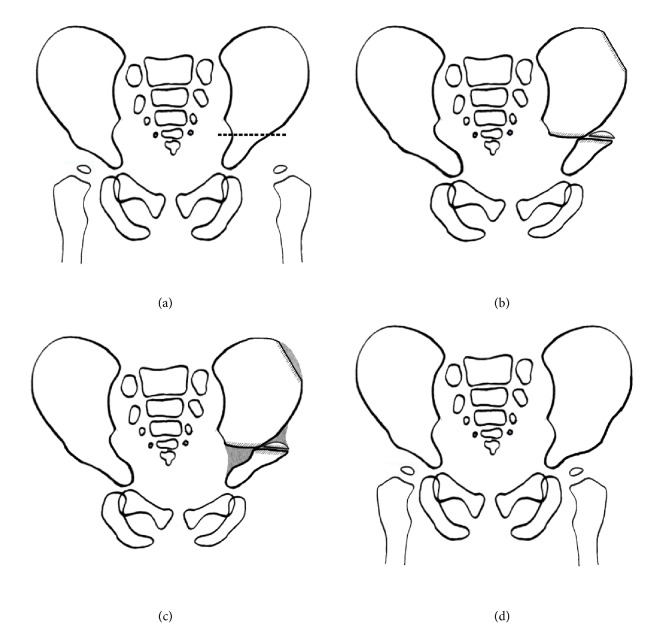
(a) The osteotomy was performed from sciatic notch to anterior-inferior iliac spine (the dotted line). (b) The distal osteotomy segment was shifted anterolaterally. A graft was taken from the iliac wing and was inserted on the raw surface of the distal osteotomy segment of the pelvis. (c) After about one and a half months, osteogenesis (gray color) was seen in the empty spaces between the proximal and distal osteotomy segments (acetabular roofing process). (d) Completed roof of acetabulum was observed about three months after the osteotomy.

**Figure 2:**
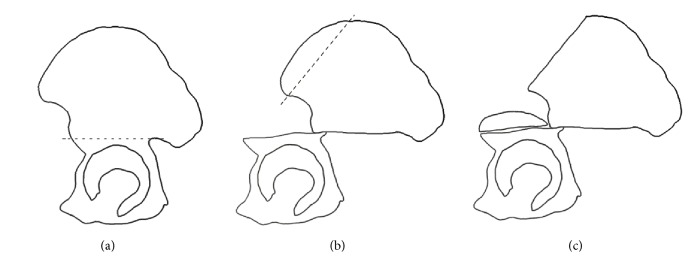
Lateral view of the modified Salter osteotomy. The distal osteotomy segment was shifted anterolaterally (going from (a) to (c)).

**Figure 3:**
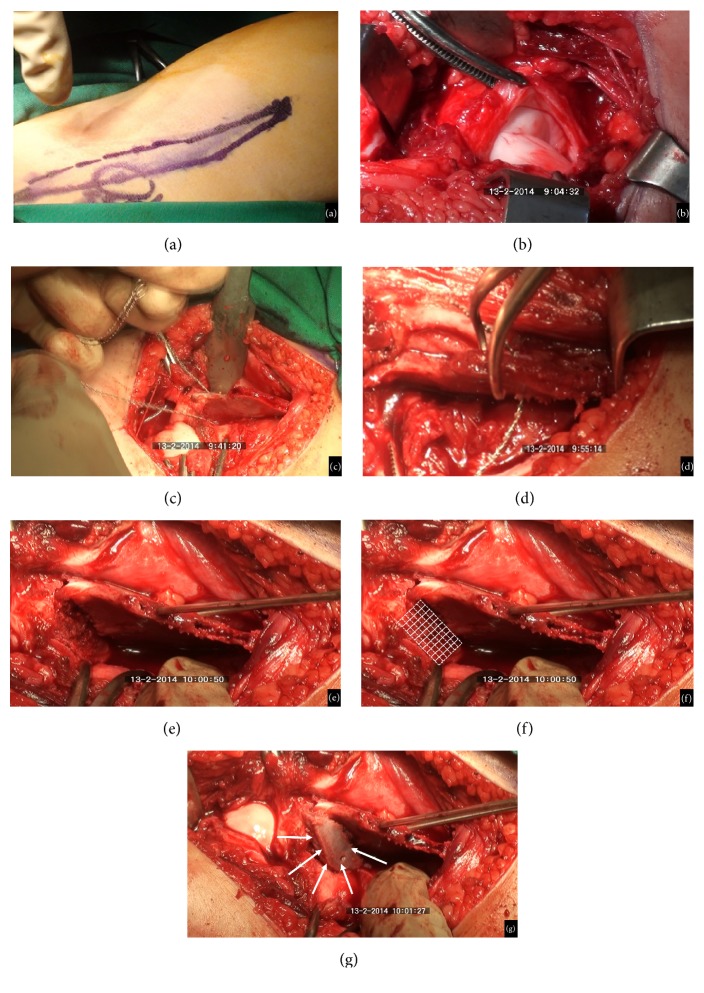
(a) Iliofemoral incision line. (b) Exposure of the hip joint and opening the capsule. (c) Pelvic osteotomy using a Gigli saw. (d) Taking a bone graft from the iliac wing. (e) Fixing the proximal and distal osteotomy segments by pins. (f) The hatched area shows the osteotomy cross-section added to the acetabular roof. (g) The bone graft was inserted on the raw surface of the distal osteotomy segment.

For the patients in which the femoral head was outside and above the acetabular fossa (the lowest point of femoral head epiphysis was at the same level or higher than the highest point of acetabular edge in the anteroposterior radiograph), before the modified Salter surgery, adductor tenotomy and iliopsoas tendon release were conducted through a medial approach. Then, skeletal traction was applied by 10-20 percent of the patient's weight from proximal of tibia of the affected limb for 1-2 weeks and simultaneously a short spica cast was applied for the normal limb to prevent pelvic tilt. Within 1-2 weeks, if the highest point of femoral head epiphysis was aligned with the triradiate cartilage, the patient underwent the open reduction and pelvic osteotomy. But if the femoral head was not successfully placed at the acetabular cavity, procedure of femoral shaft shortening was conducted in addition to the combined procedure of the open reduction and pelvic osteotomy. In a femoral shortening procedure, the hip muscles (when their origins and insertions are, respectively, proximal and distal to femoral shortening site), will be loosened and help the procedure of reduction. However, that is not enough and it is necessary to loosen those hip muscles with their origins and insertions both proximal to femoral shortening site (e.g., abductors, short external rotators of hip and some part of gluteus maximus muscle). Obviously, the femoral shortening does not loosen these muscles, and that was why we applied skeletal traction to lengthen and stretch these muscles after adductor tenotomy and iliopsoas tendon release. Figures 1–3 show the process of the modified osteotomy and the following results.

The changes in our technique compared to the classic Salter innominate osteotomy were as follows:

1. The skeletal traction was applied for the patients with the previously explained conditions.
2. Shortening of femur and its amount was conducted based on a specific criterion.
3. The distal segment of pelvic osteotomy was shifted anterolaterally, but not considerably downwards.
4. The joint capsule was not reefed in order to prevent increase in pressure to femoral head.

### 2.7. Postoperative Protocol

After 48 hours, the drain was removed. Antibiotic was administered for the first 6-7 days of surgery, including cefazolin 100 mg/kg per day intravenously every 6 hours until the first 72 hours and then it was changed to 50-60 mg/kg per day orally every 6 hours for the next 3-4 days. To control postoperative pain, acetaminophen 125 mg suppository was administered twice to three times daily.

Six weeks after the surgery, the pins were removed and the spica cast was changed to a broomstick cast in an about neutral position of abduction/adduction and rotation of the hip. At the same time, we recommended the patients to do range-of-motion exercises in all directions as much as the broomstick cast would allow. Standing and weight-bearing exercises were also recommended. After one month, the broomstick cast was removed and the patients were encouraged to begin walking. Physiotherapy was administered for 3-6 months in order to increase the hip range of motion and gait training.

### 2.8. Statistical Analysis

The collected data underwent descriptive analysis using SPSS statistical software v17. Student's t-test was used to compare the radiographic indices between different times. A p-value less than 0.05 was considered statistically significant.

### 2.9. Ethical Issues

The written informed consent was obtained from parents of the patients after a full explanation of the study. The ethics committee of Babol University of Medical Sciences (code: MUBABOL.HRI.REC.1396.93) approved this protocol. The information about each patient was kept confidential.

## 3. Results

In total, 76 patients with DDH were included in the present study, of whom 68 (89.5%) were girls and 8 (10.5%) were boys. The mean age at the time of operation was 2 years and 11 months, ranging from 1.5 to 16 years old. The duration of surgery ranged from 1 to 1.5 hours. The mean follow-up period was 4 years and one month (1 year and 3 months to 7 years and 9 months). Out of 76 subjects, 39 had left DDH and 23 had right DDH, and 14 were bilaterally affected; therefore a total of 90 hips were assessed.

Based on the modified McKay criteria, out of 90 hips evaluated, 76 (84.5%) had excellent results, 9 (10%) had good results and 5 (5.5%) had fair results at final follow-up. No patient had a poor result.

**Figure 4:**
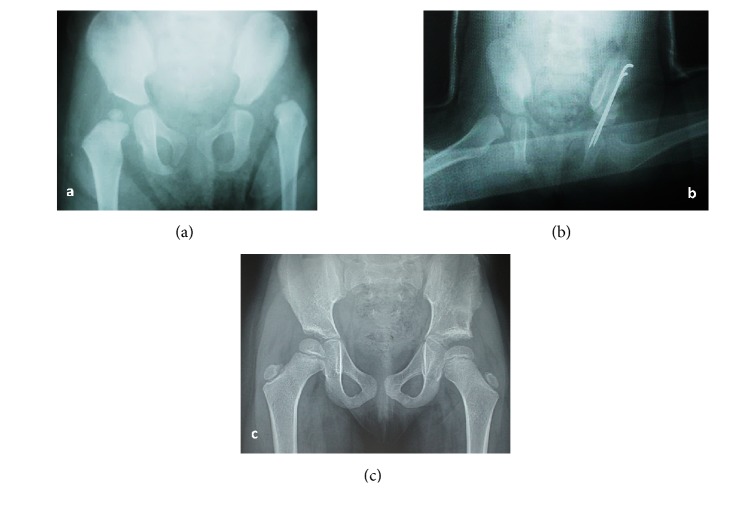
(a) Preoperative radiograph in an 18-month-old girl with left developmental dysplasia of hip. (b) Immediate postoperative radiograph. (c) Radiograph taken at 4-year follow-up.

**Figure 5:**
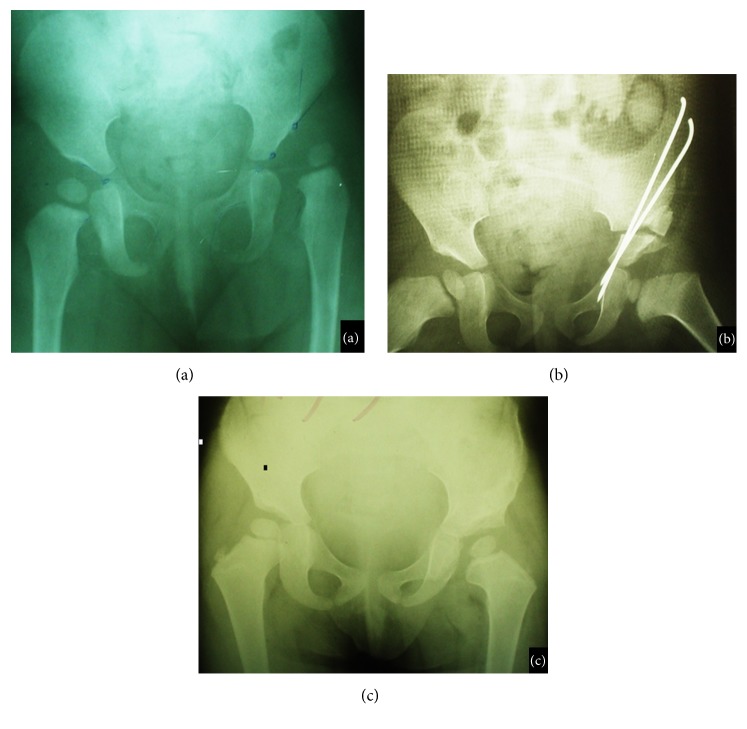
(a) Preoperative radiograph in a 23-month-old girl presetting with left developmental dysplasia of hip. (b) Immediate postoperative radiograph. (c) Radiograph taken at 3.5-year follow-up.

Based on the modified Severin criteria, 70 hips (77.8%) were grade IA, 11 (12.2%) were grade IB, 6 (6.7%) were grade II, and 3 (3.3%) were grade III at the final follow-up. The mean acetabular index before surgery was 47.85° (41° to 59). Immediately after the surgery, it was 17.16° (13° to 22°) (p<0.0001) and then reduced to 11.24° (7° to 19°) (p<0.0001) at the final follow-up. The mean initial postoperative CE angle was 30.3° (25° to 42°) and this progressed significantly to 39.1 (31° to 56°) at the final follow-up (p<0.0001). Figures 4 and 5 indicate the preoperative, immediate postoperative and follow-up radiographs taken from patients presented with DDH.

In the radiological assessments, 4 hips (4.4%) developed AVN of the femoral head, of which 3 were grade II and one was grade III as classified by Bucholz and Ogden.

## 4. Discussion

This study was conducted to evaluate the outcomes of the modified Salter innominate osteotomy on 90 hips from 76 patients. Overall, 84.5% had excellent and 94.5% had excellent and good results according to the modified McKay criteria. In the study by Bhuyan [14], who conducted open reduction, femoral shortening derotation and Salter's osteotomy on 25 patients (30 hips), the McKay's score was excellent only in 43% of hips and 89% in total had excellent and good results. In the study by Tukenmez and Tezeren [15] conducted on 79 hips, it was stated that 45.6% of the hips were in the excellent condition and 19% were not in a satisfactory condition. In comparison to the mentioned studies and others [16–18] reporting Salter's technique, our newly designed method had a higher rate of excellent results, as measured by McKay criteria.

Based on the modified Severin radiological criteria, 96.7% of our hips had satisfactory conditions. Also, the mean acetabular index was 17.16° postoperatively, significantly improving to 11.24° at the final follow-up. Besides, we witnessed a significant improvement in the mean CE angle at the final follow-up (39.1°) compared to immediate postoperative time (30.3°). Our good results were similar to some previously published data used Salter innominate method [15–17, 19] and better than others [14, 20, 21]. These findings show acceptably good outcomes for our modified technique.

These good Severin findings in our study can be explained by that the distal osteotomy segment was not shifted downwards. This method prevents pressure on the triradiate cartilage and therefore remodeling of the acetabulum is better than in the classic Salter's method, and consequently the Severin score would be better in our technique. In the Salter's method, the distal segment is shifted downwards with lateral opening wedge (which is maintained by wedge-shaped graft) when the medial ridges of the raw surfaces of the osteotomy sites are laid together and are not opened (causing plastic deformation of acetabular roof), hence, the hinge action of symphysis pubis in this condition is questionable, but hinge load is resisted by the triradiate cartilage. Therefore, in addition to the head compression in this position, the triradiate cartilage is under bending force with traction side medially and compression side laterally. These pressures in the Salter's method can affect the acetabular growth-plate and prevent the normally maximum remodeling of acetabulum, whereas in our osteotomy technique, when the distal segment of pelvic is shifted anterolaterally in the amount of osteotomy cross-section, it should be moved some circularly simultaneously, around the center of the symphysis pubis (hinging on the symphysis pubis as the center of segment rotation), and passes a soft descending arch in the amount of a portion of circle circumference with center of symphysis pubis, without pressure on triradiate cartilage which has an important role in remodeling of acetabulum.

Regarding postoperative complications, we witnessed AVN in 4 hips (4.4%) which was a low rate compared to several studies [22–25]. Graft or pin displacement, relocation of osteotomy segments, redislocation of the hip, hematoma, superficial and deep infections, persistent stiffness of the hip joint, femoral fracture, and AVN of the femoral head are examples of postoperative complications of the classic Salter osteotomy [26, 27]. We believe that skeletal traction after adductor tenotomy and iliopsoas release for 1-2 weeks (based on the above-mentioned conditions) and before open reduction and pelvic osteotomy, and also not reefing the capsule after the open reduction, avoid excessive pressure on the femoral head after the surgery, and thereby has significant effects on decrease in rate of AVN. Additionally, in our modified method, the distal segment is completely (i.e., in the amount of osteotomy cross-section) shifted only forwards and outwards, so no gap will be created, which can decrease or disappear the pressure on femoral head, risk of chondrolysis, AVN, and displacement of the segments after pinning.

One of our study limitations was related to the follow-up period which was not long enough to evaluate the long-term radiographic and functional results. However, considering that our paper was a report of a modified method, we believe that the current follow-up time was acceptable. We also propose a study to be performed assessing parallel both of the classic Salter method and our modified technique simultaneously. One of the strength points of our study was that all surgeries were conducted by the same surgeon, increasing the homogeneity of the patient group.

## 5. Conclusion

Our findings showed that our newly modified Salter osteotomy for the treatment of the patients aged ≥ 18 months presenting with DDH had less postoperative complications and better clinical and radiographic results compared to the classic innominate osteotomy. In the osteotomy of pelvic for treatment of DDH, a less trauma (tension-compression) to the acetabulum and femoral head leads to better surgery outcomes. Hence, we believe that shifting the acetabular roof downwards using lateral opening wedge-shaped graft in the classic Salter osteotomy not only presses the femoral head and decreases the acetabular volume, but also likely damages the growth-plate of acetabulum (triradiate cartilage), whereas in our modified method, we virtually do not shift the distal osteotomy segment downwards, preventing the pressure to the triradiate cartilage.

## Figures and Tables

**Table 1:** The modified McKay criteria for clinical evaluation.

Excellent	Stable, painless hip, no limp, negative Trendelenburg sign, and a full range of movement.

Good	Stable, painless hip, slight limp, negative Trendelenburg sign, and a slight decrease in range of movement.

Fair	Stable, painless hip, limp, positive Trendelenburg sign, and limitation of movement.

Poor	Unstable or painful hip, or both; positive Trendelenburg sign.

**Table 2:** The modified Severin classification for radiographic results.

Excellent	IA	CE*∗* angle > 19°, age 6 to 13 years; CE angle > 25°, age > 14 years.

Good	IB	CE angle 15° to 19°, age 6 to 13 years; CE angle 20° to 25°, age > 14 years.

	II	Moderate deformity of femoral head, femoral neck or acetabulum but otherwise same as grade I.

Fair	III	Dysplastic hip, no subluxation; CE angle < 20°, age > 14 years.

Poor	IV	Subluxation.

	V	Femoral head in false acetabulum.

	VI	Redislocation.

*∗* CE, center-edge.

## Data Availability

The data used to support the findings of this study are available from the corresponding author upon request.

## References

[1] Noordin S., Umer M., Hafeez K., Nawaz H. (2010). Developmental dysplasia of the hip. *Orthopedic Reviews*.

[2] Bracken J., Tran T., Ditchfield M. (2012). Developmental dysplasia of the hip: Controversies and current concepts. *Journal of Paediatrics and Child Health*.

[3] Schwend R. M., Shaw B. A., Segal L. S. (2014). Evaluation and treatment of developmental hip dysplasia in the newborn and infant. *Pediatric Clinics of North America*.

[4] Shorter D., Hong T., Osborn D. A. (2013). Cochrane Review: Screening programmes for developmental dysplasia of the hip in newborn infants. *Evidence-Based Child Health*.

[5] Emara K., Kersh M. A. A., Hayyawi F. A. (2018). Duration of immobilization after developmental dysplasia of the hip and open reduction surgery. *International Orthopaedics*.

[6] Gulati V., Eseonu K., Sayani J. (2013). Developmental dysplasia of the hip in the newborn: A systematic review. *World Journal of Orthopedics*.

[7] Thomas S. (2015). A review of long-term outcomes for late presenting developmental hip dysplasia. *The Bone & Joint Journal*.

[8] Salter R. B. (1961). Innominate osteotomy in the treatment of congenital dislocation and subluxation of the hip. *Journal of Bone and Joint Surgery*.

[9] De Pellegrin M., Moharamzadeh D. (2010). Developmental dysplasia of the hip in twins: The importance of mechanical factors in the etiology of DDH. *Journal of Pediatric Orthopaedics*.

[10] Gür E., Sarlak O. (1990). The complications of salter innominate osteotomy in the treatment of congenital dislocation of hip. *Acta Orthopædica Belgica*.

[11] Barrett W. P., Staheli L. T., Chew D. E. (1986). The effectiveness of the Salter innominate osteotomy in the treatment of congenital dislocation of the hip. *The Journal of Bone and Joint Surgery*.

[12] Severin E. (1941). Contribution to the knowledge of congenital dislocation of the hip joint. *Acta Chirurgica Scandinavica*.

[13] Bucholz R., Ogden J. Patterns of ischemic necrosis of the proximal femur in nonoperatively treated congenital hip disease.

[14] Bhuyan B. K. (2012). Outcome of one-stage treatment of developmental dysplasia of hip in older children. *Indian Journal of Orthopaedics*.

[15] Tukenmez M., Tezeren G. (2007). Salter innominate osteotomy for treatment of developmental dysplasia of the hip. *Journal of Orthopaedic Surgery*.

[16] Chen Q., Deng Y., Fang B. (2015). Outcome of one-stage surgical treatment of developmental dysplasia of the hip in children from 1.5 to 6 years old. A retrospective study. *Acta Orthopaedica Belgica*.

[17] da Rocha V. L., Marques G. L., da Silva L. J. (2014). Clinical and radiographic medium‐term evaluation on patients with developmental dysplasia of the hip, who were submitted to open reduction, capsuloplasty and Salter osteotomy. *Revista Brasileira de Ortopedia*.

[18] Abdullah E. A., Razzak M. Y., Hussein H. T., El-Adwar K. L., Abdel-RazekYoussef A. (2012). Evaluation of the results of operative treatment of hip dysplasia in children after the walking age. *Alexandria Journal of Medicine*.

[19] Chang C.-H., Kao H.-K., Yang W.-E., Shih C.-H. (2011). Surgical results and complications of developmental dysplasia of the hip–one stage open reduction and Salter’s osteotomy for patients between 1 and 3 years old. *Chang Gung Medical Journal*.

[20] Macnicol M. F., Bertol P. (2005). The Salter innominate osteotomy: Should it be combined with concurrent open reduction?. *Journal of Pediatric Orthopaedics B*.

[21] Kotzias Neto A., Ferraz A., Bayer Foresti F., Barreiros Hoffmann R. (2014). Bilateral developmental dysplasia of the hip treated with open reduction and Salter osteotomy: Analysis on the radiographic results. *Revista Brasileira de Ortopedia*.

[22] Gulman B., Tuncay L. C., Dabak N., Karaismailoglu N. (1994). Salter’s innominate osteotomy in the treatment of congenital hip dislocation: A long-term review. *Journal of Pediatric Orthopaedics*.

[23] Baghdadi T., Bagheri N., Kalantar S. H., Khabiri S. S. (2018). The outcome of salter innominate osteotomy for developmental hip dysplasia before and after 3 years old. *The Archives of Bone and Joint Surgery*.

[24] Sadeghpour A., Rouhani A., Mohseni M. A., Aghdam O. A., Goldust M. (2012). Evaluation of surgical treatment of developmental dysplasia of hip for avascular necrosis of femoral head in children. *Pakistan Journal of Biological Sciences*.

[25] Böhm P., Brzuske A. (2002). Salter innominate osteotomy for the treatment of developmental dysplasia of the hip in children: Results of seventy-three consecutive osteotomies after twenty-six to thirty-five years of follow-up. *The Journal of Bone & Joint Surgery*.

[26] Kotlarsky P., Haber R., Bialik V., Eidelman M. (2015). Developmental dysplasia of the hip: What has changed in the last 20 years?. *World Journal of Orthopedics*.

[27] Ezirmik N., Yildiz K. (2011). A study on the complications of surgical treatment for bilateral developmental dysplasia of the hip and a comparison of two osteotomy techniques. *The Eurasian Journal of Medicine*.

